# An integrated platform for bovine DNA methylome analysis suitable for small samples

**DOI:** 10.1186/1471-2164-15-451

**Published:** 2014-06-09

**Authors:** Habib A Shojaei Saadi, Alan M O’Doherty, Dominic Gagné, Éric Fournier, Jason R Grant, Marc-André Sirard, Claude Robert

**Affiliations:** Laboratory of Functional Genomics of Early Embryonic Development, Institut des nutraceutiques et des aliments fonctionnels, Faculté des sciences de l’agriculture et de l’alimentation, Pavillon des services, Université Laval, Québec, G1V 0A6 Canada; School of Agriculture, Food Science & Veterinary Medicine, University College Dublin, Belfield, Dublin, Ireland; Department of Agricultural, Food and Nutritional Science, University of Alberta, Edmonton, AB T6G 2P5 Canada

**Keywords:** Epigenome, DNA methylation, Bovine embryo, Methylome and transcriptome parallel analysis, Analysis pipeline, CpG enrichment, Repetitive elements, Epigenome-wide association study

## Abstract

**Background:**

Oocytes and early embryos contain minute amounts of DNA, RNA and proteins, making the study of early mammalian development highly challenging. The study of the embryo epigenome, in particular the DNA methylome, has been made accessible thanks to the possibility of amplifying specific sequences according to their initial methylation status. This paper describes a novel platform dedicated to the genome-wide study of bovine DNA methylation, including a complete pipeline for data analysis and visualization. The platform allows processing and integrating of DNA methylome and transcriptome data from the same sample. Procedures were optimized for genome-wide analysis of 10 ng of DNA (10 bovine blastocysts). Bovine sperm and blastocysts were compared as a test of platform capability.

**Results:**

The hypermethylation of bovine sperm DNA compared to the embryo genome was confirmed. Differentially methylated regions were distributed across various classes of bovine sperm genomic feature including primarily promoter, intronic and exonic regions, non-CpG-island regions (shore, shelf and open-sea) and CpG islands with low-to-intermediate CpG density. The blastocyst genome bore more methylation marks than sperm DNA only in CpG islands with high CpG density. Long-terminal-repeat retrotransposons (LTR), LINE and SINE were more methylated in sperm DNA, as were low-complexity repetitive elements in blastocysts.

**Conclusions:**

This is the first early embryo compatible genome-wide epigenetics platform for bovine. Such platforms should improve the study of the potential epigenetic risks of assisted reproductive technologies (ART), the establishment sequence of embryonic cell lines and potential deviations in both gene expression and DNA methylation capable of having long-term impact.

**Electronic supplementary material:**

The online version of this article (doi:10.1186/1471-2164-15-451) contains supplementary material, which is available to authorized users.

## Background

The study of early embryonic development continues to pose formidable technical challenges due in large part to the limited amounts of sample material. However, high-throughput high-fidelity amplification of nucleic acid is making the macromolecular study of embryonic physiology more accessible. Microarray platforms, and more recently RNAseq, have made studying the early embryo transcriptome almost routine. Our group has been developing bovine and porcine microarrays based on transcriptomic platforms that include standardized sample preparation procedures and a complete user-friendly software suite for data normalization and analysis, allowing efficient processing of samples from extraction through to the generation of publishable graphs [1, 2]. Transcriptomic platforms have been used to study how early embryos of many different species interact with their immediate microenvironment [3–8]. Although very useful, the transcriptome has not allowed us to determine whether or not deviant gene expression, that is observed in embryos, is a transient adaptation to surrounding conditions that later yields to normal expression without any long-term impact on development. In order to provide a more complete picture of embryo adaptation and its potential long-term consequences, study of the epigenome is necessary [9, 10]. The epigenome is the sum of all epigenetic information [11] and refers more precisely to the complete description of chemical changes to DNA and histones [12], including histone tail modifications, chromatin remodelling proteins, and ncRNA. Epigenomics and transcriptomics are closely interrelated in terms of gene function and regulation [13, 14] and together modulate gene expression.

Among epigenomic effectors, DNA methylation is believed to be a strong primary molecular mark having a major impact on intergenerational gene silencing [15, 16]. DNA methylation patterns are known to be relatively stable and established in a tissue-specific manner [17, 18]. However, following fertilization and during mammalian pre-implantation development, the DNA methylation pattern is dynamic and undergoes reprogramming in the form of a wave of genome-wide de-methylation and re-methylation [19–24], thus putting the embryo at risk of programming errors [25]. Furthermore, the study of how the DNA methylome can be modified by changes in the embryo microenvironment such as *in vivo/vitro* culture, uterine conditions, or maternal nutritional regimen has represented a major challenge and continues to do so, due mainly to sample scarcity offering input DNA well below minimal recommendations.

Numerous platforms already exist to study methylation of targeted loci or to obtain genome-wide methylation profiles. For the study of very small samples, determining DNA methylation at targeted loci has so far been more successful than genome-wide approaches [26]. The main advantages of general survey are the possibility of describing physiological responses at the genome-wide scale and the potential for novel discovery.

The aim of the present work was to develop a technological platform that is complementary to existing platforms, in order to provide a whole-genome view of DNA methylation in bovine early embryos. Since a diploid mammalian nucleus contains about 6.8 picograms of DNA (http://www.genomesize.com) and the expanded blastocyst of large mammalian species is composed of about 150 cells, a single bovine blastocyst contains approximately 1 ng of DNA. The current benchmark for minimal sample size is around 10 ng, therefore corresponding to a pool of 10 expanded blastocysts. The other criterion is ease of use, at both the sample handling and data processing steps. We thus sought to identify an existing methodological approach which would be best suited to analyze very small samples of DNA. The platform was tested and validated using experimental samples.

## Results

### Platform design

**Figure 1:**
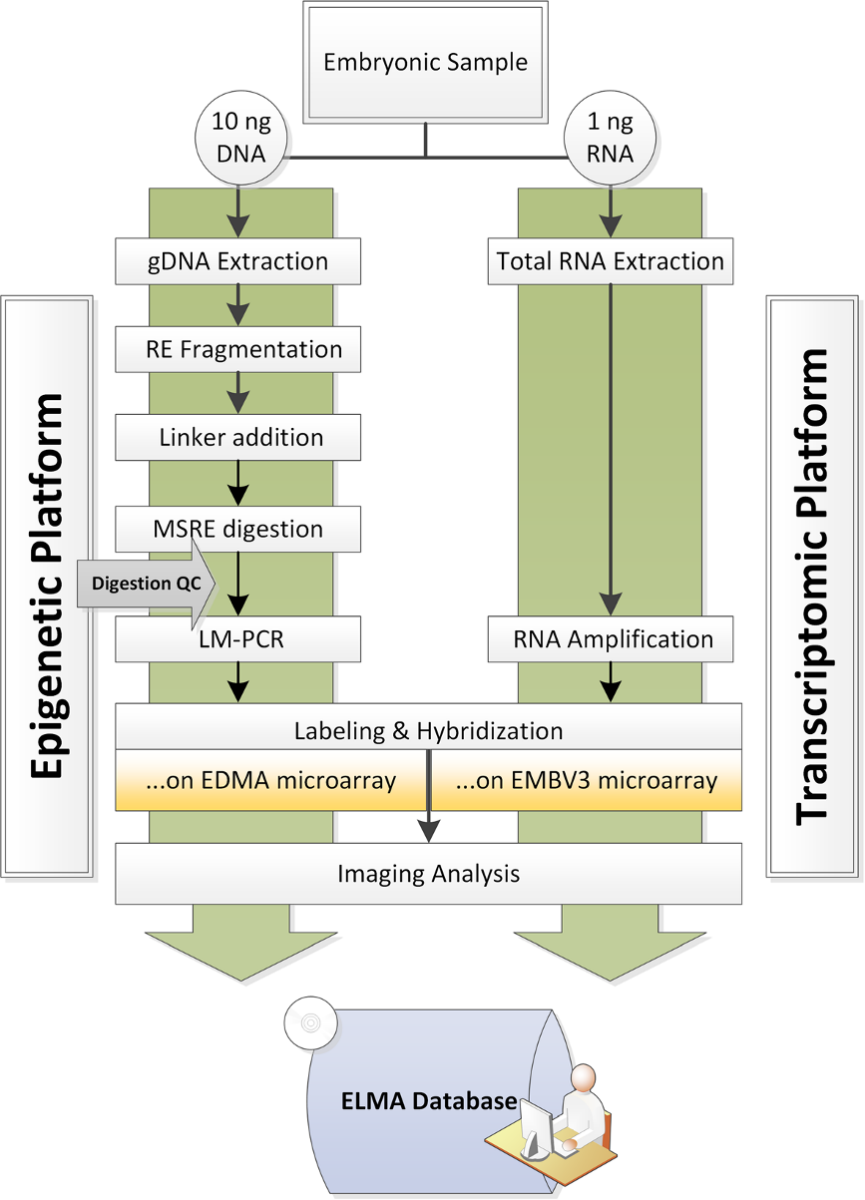
**Sequence of steps involved in the generation of methylome and transcriptome data from the same sample using EDMA platform.** A quality control step prior to LM-PCR allows evaluation of the efficiency of cleavage by MSREs for methylome analysis. **EDMA:** EmbryoGENE DNA methylation analysis platform, **ELMA:** EmbryoGENE LIMS and Microarray Analysis, **EMBV3:** EmbryoGENE bovine transcriptomics microarray Version 3, **LM-PCR:** Ligation-mediated PCR, **MSRE:** Methyl-sensitive restriction endonuclease, **RE:** Restriction endonuclease (*MseI)*.

The EmbryoGENE (http://embryogene.ca) DNA Methylation Analysis (EDMA) platform was designed for high-throughput methylation profiling of bovine genome using limited amounts of input material. It combines four independent methodological principles: i) restriction endonuclease-based (RE) (*MseI*) genomic DNA fragmentation; ii) targeting methylated regions using a cocktail of methyl-sensitive restriction endonucleases; iii) amplification of methylated (thus protected) fragments using ligation-mediated PCR; and iv) identification of the amplified methylated fragments using a microarray. The EDMA workflow is presented in Figure 1.

**Figure 2:**
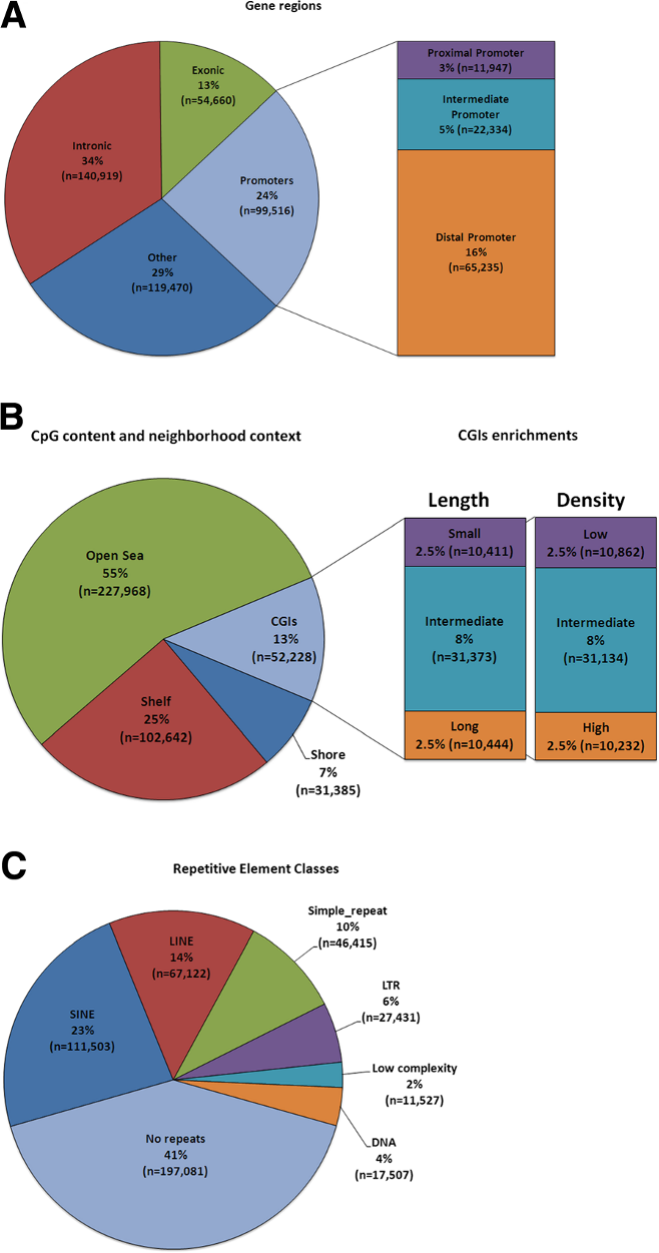
**The characteristics of EDMA array. (A)** Gene region coverage by the probes. The single greatest proportion (34%) corresponds to intronic regions. **(B)** Probe distribution based on proximity to CpG islands as well as CpG islands-related enrichments. More than half of the probes target fragments in the open-sea region. **(C)** Proportions of different classes of bovine repetitive elements detectable by the EDMA platform.

**Figure 3:**
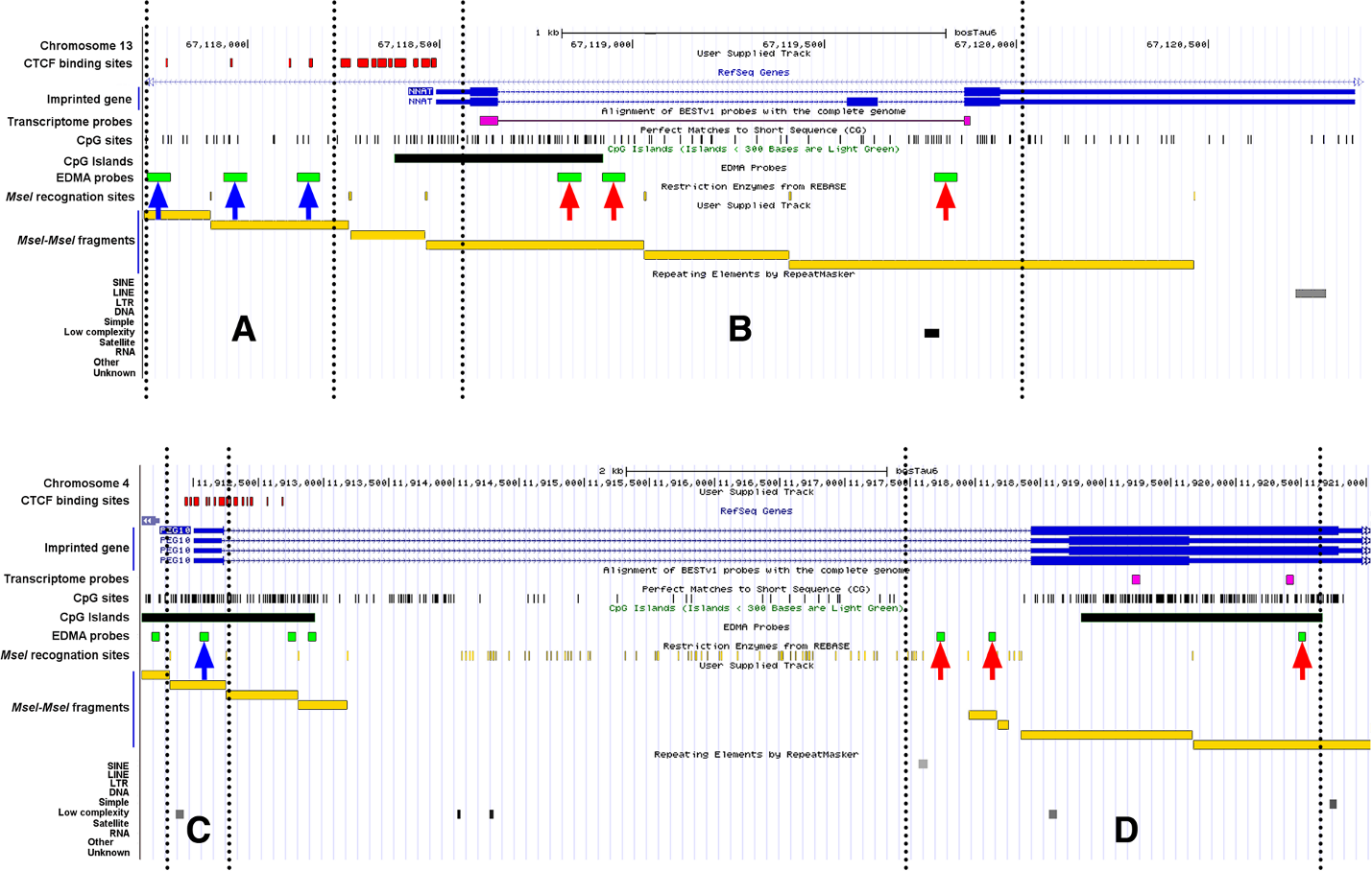
**Snapshots from the UCSC genomic web-browser describing the genomic locations of two bovine imprinted genes (NNAT and PEG10), positioning of the probes (methylome and transcriptome) and other associated bovine genomic features.** *MseI* recognition sites on genomic DNA result in *MseI-MseI* fragments. CpG islands may be absent (Area **A)** or present (Areas **B-D)**. EDMA probes may target CTCF binding sites (blue arrows), which may be proximal (Area **A**) or within (Area **C)** the gene body. Other EDMA probes (red arrows) also may overlap with probes the transcriptomic (EMBV3) array or cover CpG islands or non-CpG islands. Probes (red arrows) may also cover intronic or exonic portions of the genome (Area **D)**.

Sample treatment protocol and microarray design were optimized in parallel. Our laboratory has previously conducted a survey of DNA methylation in bovine embryos using various reduced representation approaches [27]. This allowed us to identify a collection of loci at which DNA methylation varies in association with early development. These loci included CpG islands, gene bodies, intergenic regions and repetitive elements. The oligo design accounted for the sample preparation steps in which genomic DNA was fragmented using the *MseI* restriction enzyme, which recognizes 5′-T/TAA-3’, thus avoiding methylated cytosine residues. *In silico* digestion of the bovine genome shows that *MseI* yields fragments averaging 160 bp in length (see Additional file 1: Figure S1 A). A second layer of *in silico* analysis located the methyl-sensitive restriction endonucleases (MSRE) restriction sites, namely C/CGG (*HpaII*), GC/GC (*HinP1I*) and C/CGC (*Aci1I*). Distribution of common and unique MSRE sites within *MseI* fragments is shown in Additional file 1: Figure S1 B. Furthermore, the information regarding the number of CpG sites per restriction fragments and the number of MSREs restriction sites per restriction fragments are provided in Additional file 1: Figure S2 A-D. The 60-mer oligo design was based, in part, on a collection of *MseI* fragments containing MSRE sites within the genomic loci that we previously found to bear methylation or hydroxymethylation marks in early bovine embryos [27], to which were added CpG islands determined by *in silico* analysis. Additional oligos were designed by tiling the *MseI* fragments adjacent to this initial set of targets until the capacity of a single microarray slide was reached (1×1 M oligos). Preliminary hybridizations allowed selection of a subset of 400 K oligos that performed well, based on sequence specificity and signal strength variations across the set of test hybridizations (data not shown). The final probe collection queries a variety of different genomic features not limited to CpG islands. A summary of the genomic targets surveyed by the microarray is shown in Figure 2 and Additional file 2: Table S2-4. As illustrated for two bovine imprinted genes (NNAT, PEG10) in Figure 3, the EDMA probes were distributed across various genomic features (intergenic, promoters, gene body, and repetitive elements).

**Figure 4:**
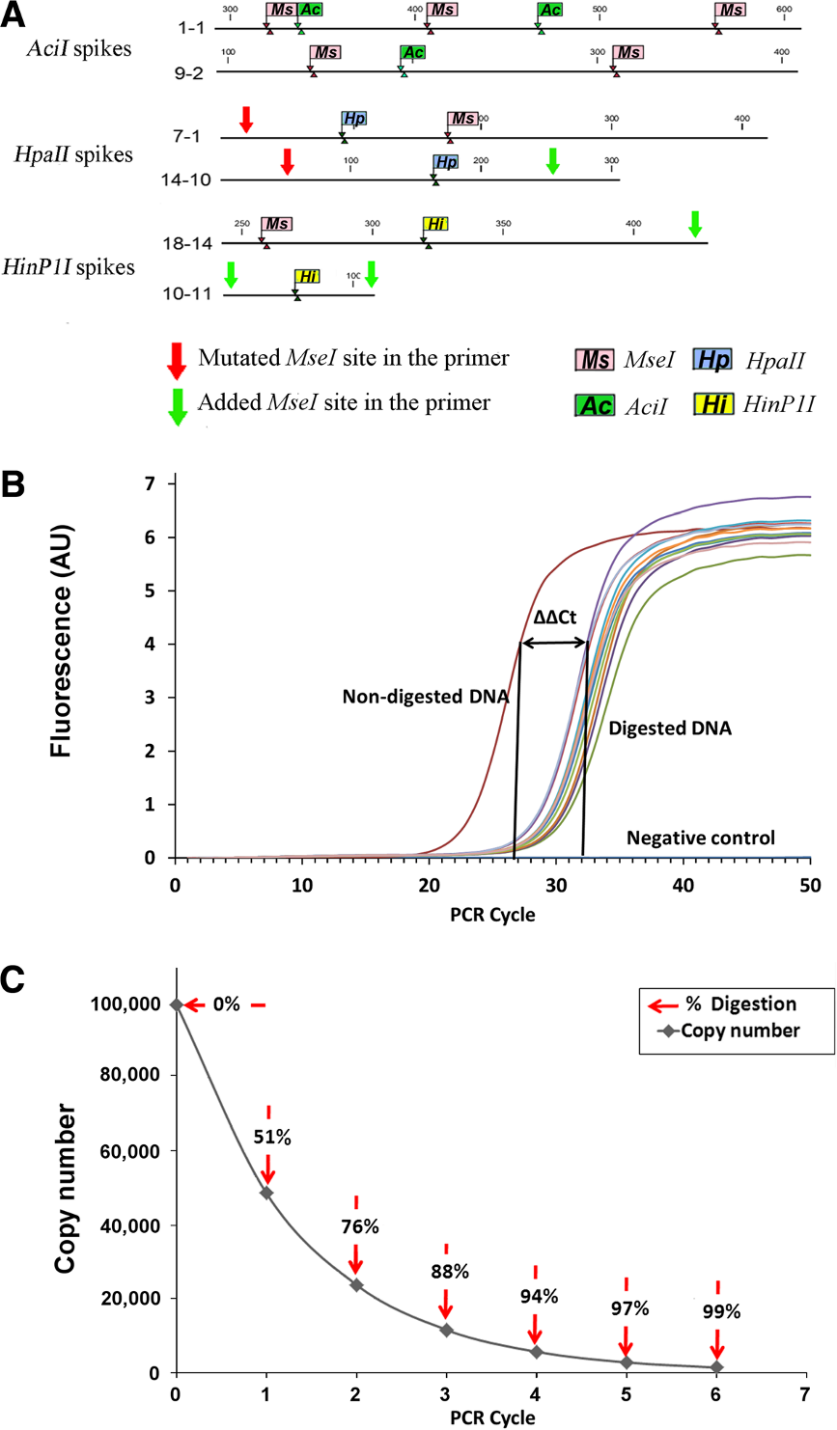
**The feature of EDMA spiked-in controls and the quality control process of MSREs digestion assessment. (A)** Location of sites cleaved by endonucleases within exogeous control DNA. **(B)** Difference in PCR threshold cycle (ΔΔCt) between un-cleaved (positive control) and cleaved samples. **(C)** Plot of residual number of copies and corresponding efficiency of cleavage by MSRE versus PCR cycle number, indicating that satisfactory cleavage (97%) is reached at the end of five PCR cycles.

Since the platform uses a cocktail of MSREs to target genomic locations bearing methylation marks, the extent of cleavage is a critical factor. Incomplete cleavage will lead to false positive results. For quality control purposes, control DNA templates were designed to account for extent of cleavage both by *MseI* and the MSREs cocktail. A pair of DNA templates was designed for each MSRE (Figure 4A). All spiked-in controls had internal *MseI* sites at their ends, and for each pair, one template was methylated *in vitro* for protection against MSRE activity. The controls were thus subjected to the same cleavage conditions as the sample. Following genomic fragmentation, adaptor ligation and MSRE treatment, sample quality was determined using qPCR, with calculation of the extent of cleavage for each MSRE. The difference in threshold cycle (C_t_) between protected (hypermethylated) and unprotected (hypo/unmethylated) control templates can be calculated, while sample uniformity can be visualized from the amplification curves (Figure 4B, 4C). Only samples displaying uniformity with the other samples cleaved > 97% in the same cohort were retained for downstream treatment. Insufficiently cleaved samples were in some cases subjected to a second treatment with additional MSRE digestion.

**Figure 5:**
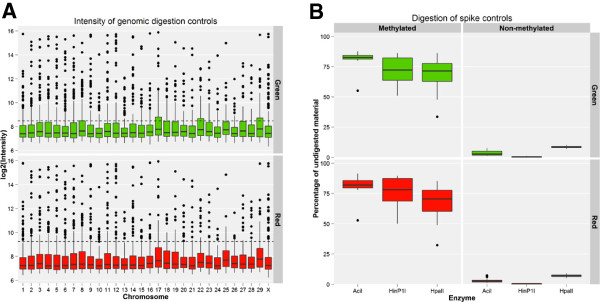
**The two types of quality control (QC) plots generating after EDMA microarray hybridization and data analysis. (A)** Signal intensity of control probes designed to overlap an *MseI* site. Probes corresponded to loci distributed throughout the bovine genome. The dashed horizontal line represents the limit of detection (mean intensity of negative controls plus four standard deviations). Intensities below this line indicate successful genomic fragmentation by *MseI*. **(B)** Cleavage of synthetic spiked-in control DNA pairs by MSRE, based on microarray signal. Signals from unprotected (unmethylated) fragments (right) are near background values.

In EDMA platform, after hybridization on the microarray and data analysis there are two types of quality control (QC) plots which the examples are shown in Figure 5. The first plot, which assesses the completeness of the genomic digestion, shows nearly complete cleavage by *MseI*, with most control probes showing signal below the background noise (Figure 5A). The second quality control assess the quality of detected signals. The plot uses signals from probes corresponding to the spiked-in controls, and confirms that unprotected templates led to low signals and vice versa for protected fragments (Figure 5B).

### The analysis pipeline

**Figure 6:**
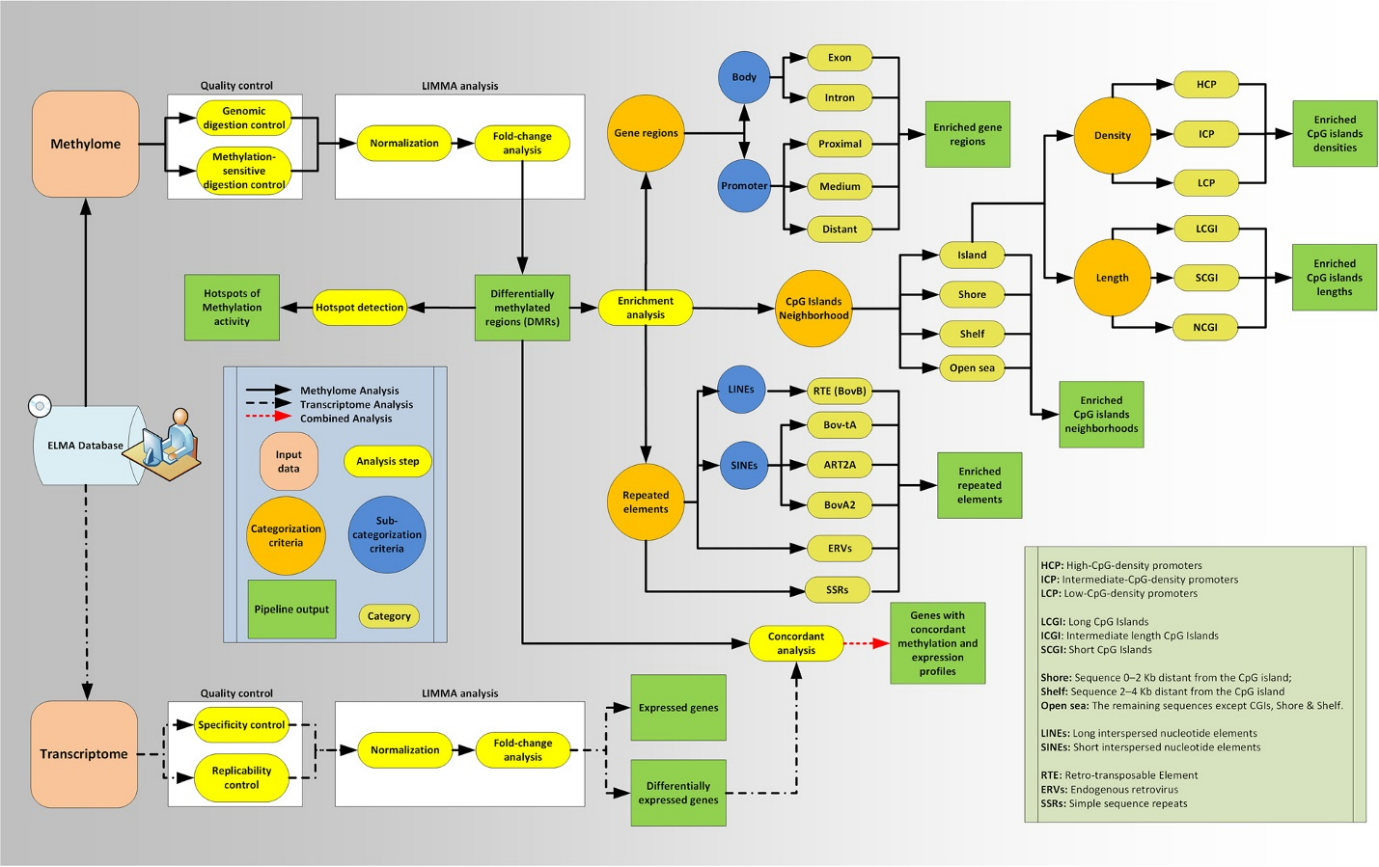
**The EDMA DNA methylation analysis pipeline.** This genome-wide methylome analysis pipeline was designed to provide a comprehensive set of plots to ease interpretation of both methylome and transcriptome raw data generated from the same sample. Information is binned according to known functions or genetic features.

**Figure 7:**
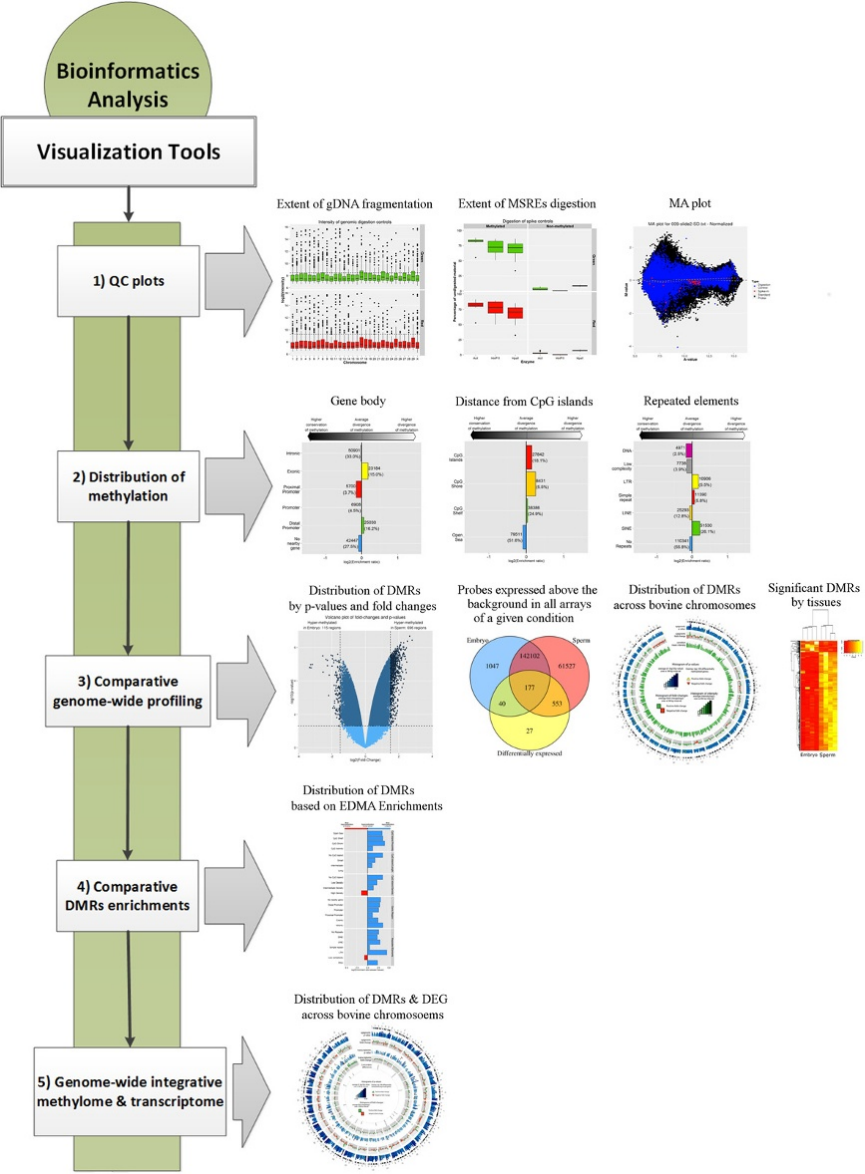
**List of the graphs generated by EDMA analytical pipeline.**

For each dye-swaps microarray hybridization, the quantification of methylation measurements are based on *M* values (the log differential- expression ratio of the two channels) which further normalized (inter & intra-array) and statistical analysis is performed as described in methods. This means larger values represented more evidence of relative methylation similar to other microarray-based genome-wide DNA methylation analysis platforms [28]. To support data mining, an extensive analysis pipeline was designed with the goal to sort the data according to defined structural characteristics (*e.g.* near known genes, within gene body components, distance from CpG islands, etc.). We designed a user-friendly and comprehensive bioinformatics data analysis pipeline to complement our developed platform (EDMA). A complete schematic of our data analysis pipeline is shown in Figure 6. The data analysis pipeline was designed to be compatible with results obtaining from our array-based gene expression platform [1]. It enables to identify alterations of DNA methylation in bovine genomic regions under various enrichment outputs in parallel to deviations in transcription. The pipeline comprises several analysis steps for data QC and differential analysis, generating a list of DMRs as well as downstream sequence-based enrichment analyses, Hot spot detection and concordant analysis in search of loci where both transcriptomic and DNA methylation are affected. The information is binned according to the set of annotations given to every genomic locus (see methods for details). Typical enrichment analysis account for CpG islands neighbourhood both in length and density, gene body structures (promoter, exons, introns) and classes of bovine repetitive elements (low complexity, SINE or LINE elements). Since EDMA is not based on bisulfite conversion and sequencing, it does not provide a relative value of the extent of DNA methylation. This information needs to be determined by targeted pyrosequening of the regions of interest.The data analysis pipeline output is a large contingent of plots that serve five different objectives (Figure 7): i) to document the quality of the samples through the quality control plots ; ii) to provide visual aid to determine where the MSRE-protected fragments were found for each sample type; iii) to provide an overall perspective of the extent of the differences in methylation between the samples; iv) to mine differentially methylated regions (DMRs) data according to genomic features; v) and lastly, the Circos plot which can be used to integrate the epigenomic and transcriptomic data (or present a chromosomal overview of either type alone).

### DNA methylation profiles of bovine sperm and early embryos

- Genome-wide overview of DNA methylation profiles.

**Figure 8:**
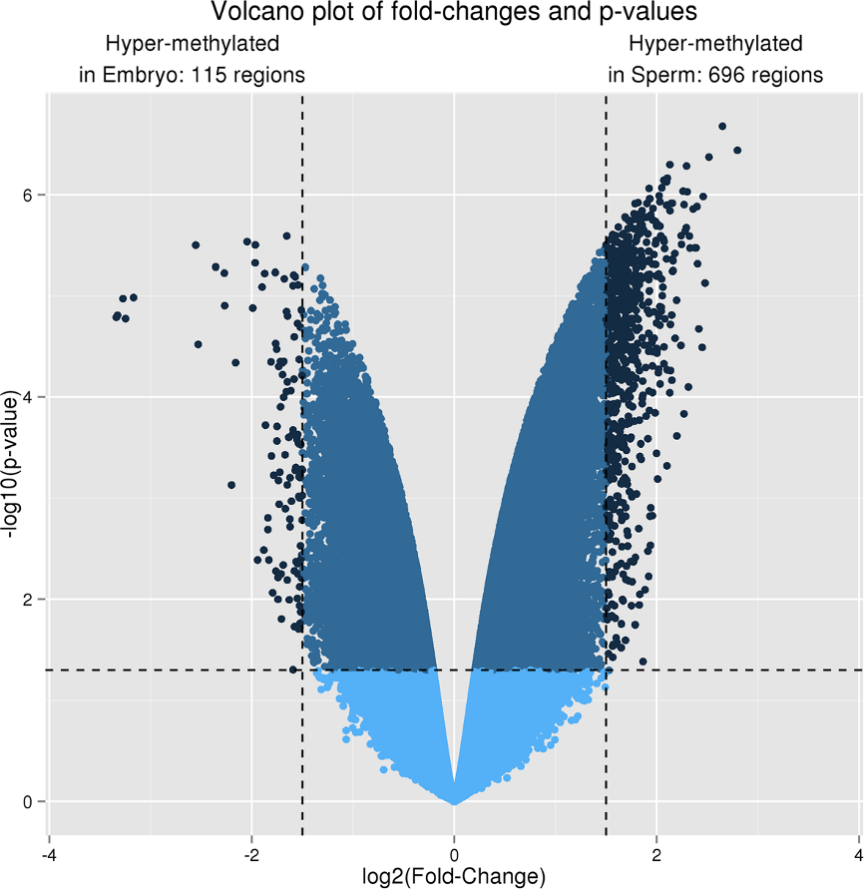
**Volcano plot of genome-wide DNA methylation.** This plot shows clearly that bovine sperm DNA was more methylated than embryo DNA.

**Figure 9:**
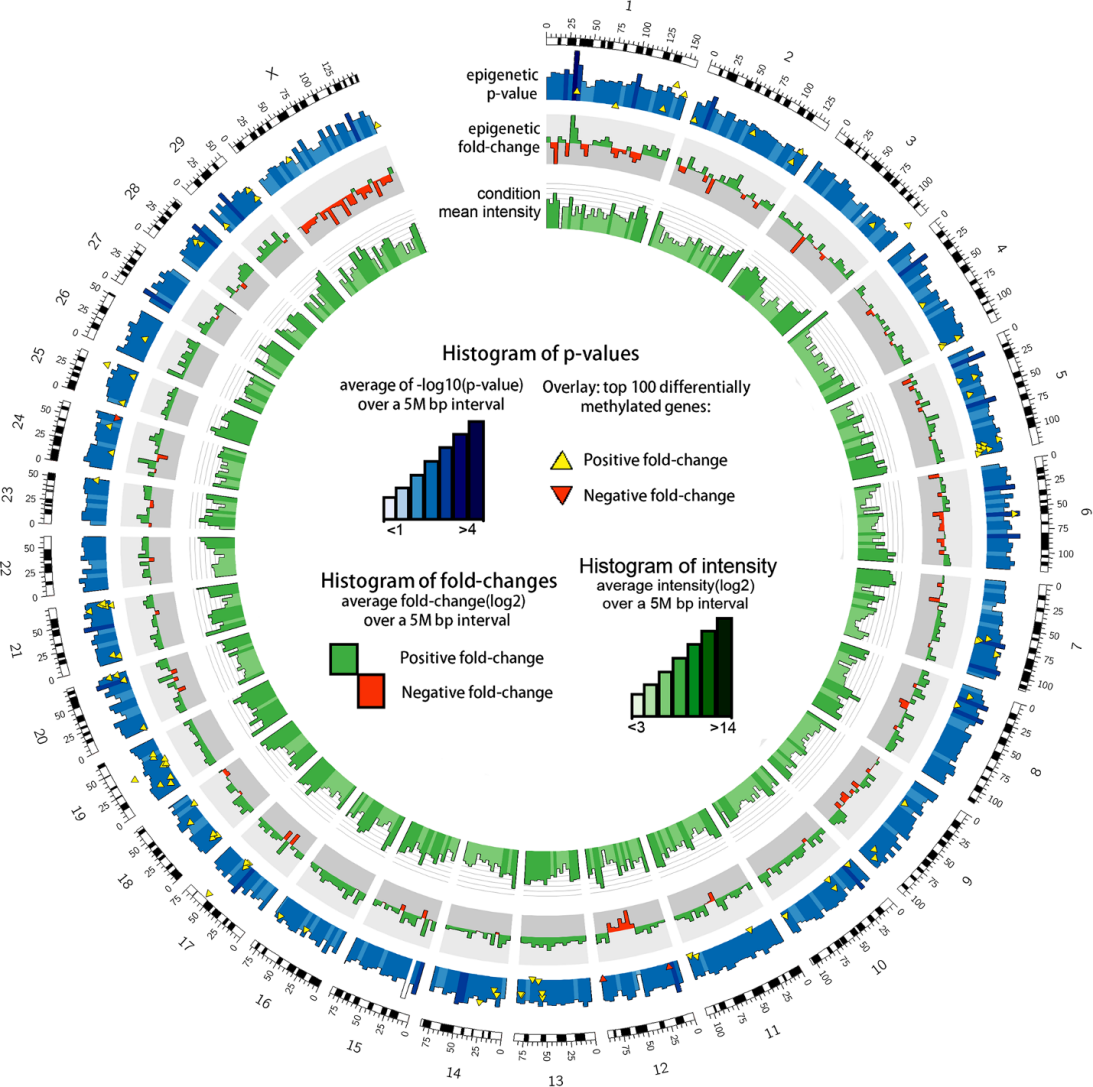
**The Circos plot showing the genome-wide DNA methylation profile of bovine sperm DNA and the blastocyst genome, by chromosome.** The mean p-values of 5 M bp windows are displayed along with the 100 most significant DMRs. Positive fold-changes represent hypermethylation in sperm while negative fold-changes represent hypermethylation in blastocysts. The inner circle depicts probe mean values across treatments to show the completeness of the coverage generated from the microarray signals.

Platform performance was evaluated on the basis of the contrast in genomic DNA methylation patterns between bovine sperm and blastocysts. Similar amounts of input DNA from both sample types were processed, and microarray data was analyzed using the pipeline described above. Using a predetermined significance criterion *i.e.* Fold-change > 2^1.5, p-value < 0.05, 811 DMRs were identified, most of which (>85%) were due to hypermethylation in sperm (Figure 8). Large numbers of the methylated loci found in the embryo genome were also found in sperm DNA. Differences can also be observed using the genome-wide view provided by Circos plot, which shows a prevalence of hypomethylation in the sperm for chromosome X compared to blastocysts DNA. Nearly all (97%) of the top 100 identified differentially methylated regions (DMRs) were found to be hypermethylated in sperm DNA (Figure 9). This reveals that the majority of DMR genes in bovine sperm DNA lose their methylation after fertilization and during early embryo development, at least until the blastocyst stage.

- Characteristics of differentially methylated regions (DMRs).

**Figure 10:**
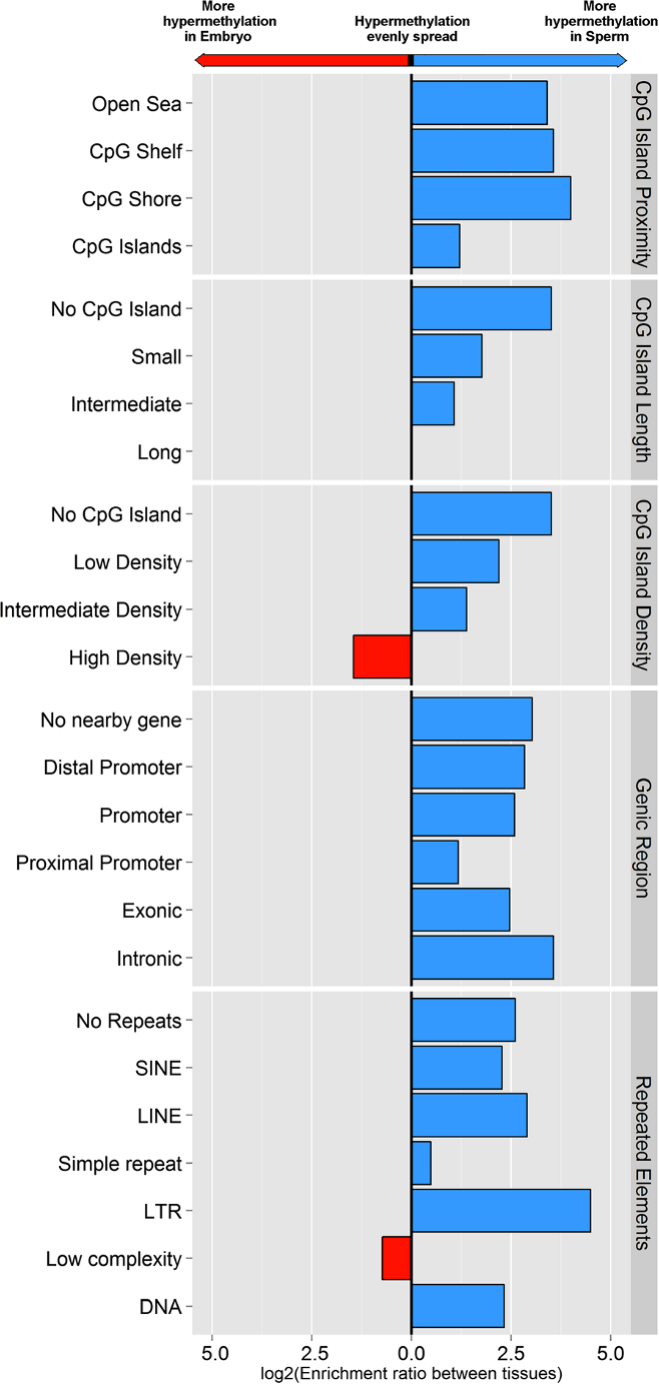
**The comparative analysis of differentially methylated regions (DMRs) enrichments between bovine sperm and blastocysts.** This figure shows the log2 enrichment ratios between the numbers of the DMRs found to be hypermethylated in bovine sperm compared to the number of DMRs found to be hypermethylated in bovine blastocysts, broken down by different types of genomic features. The majority of the DMRs showed hypermethylation in bovine sperm DNA.

Our data revealed that sperm DNA shows a tendency to hypermethylation compared to blastocysts in all types of promoter, intronic and exonic regions, non-CpG islands regions (shore, shelf and open sea) and CpG islands with low-to-intermediate CpG density and small-to-intermediate length (Figure 10). Only in high-density CpG islands in the blastocyst genome demonstrated a tendency to hypermethylation compared to sperm. Amongst DMRs containing repetitive elements, bovine sperm DNA tended to be hypermethylated in the majority of the repetitive element classes, in particular long-terminal-repeat (LTR) retrotransposons, LINE and SINE. However, low-complexity repetitive elements showed more hypermethylated in the blastocyst genome.

### DMR validation

**Figure 11:**
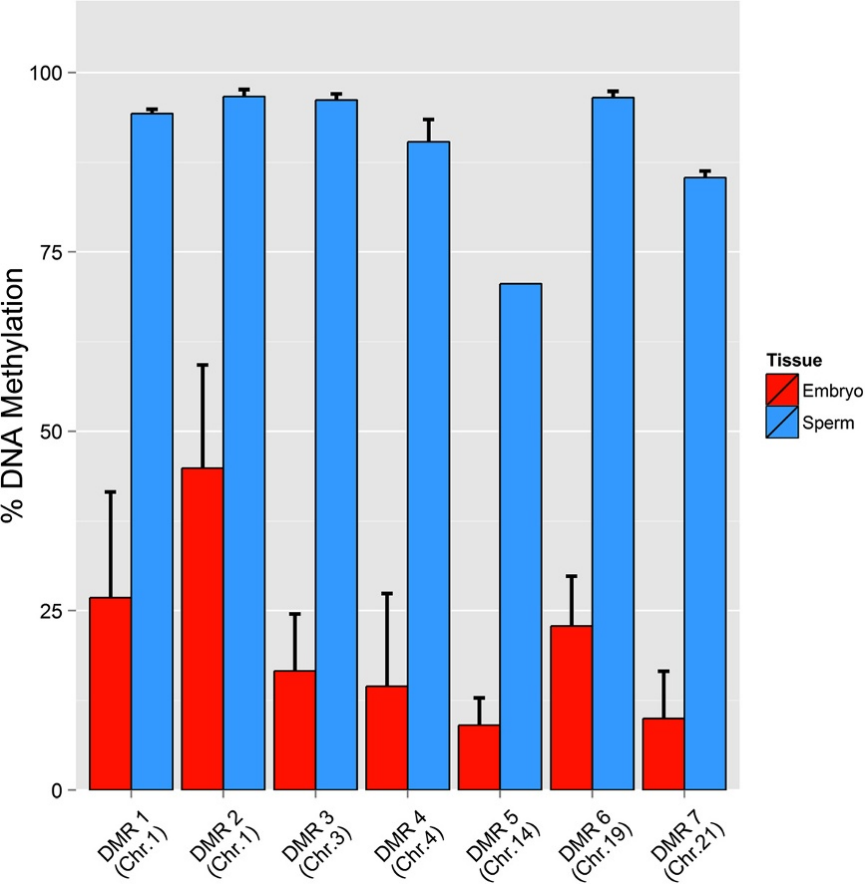
**Validation of the selected DMRs by pyrosequencing.**

Seven candidate DMR loci from different regions of the bovine genome were selected and primers were designed (see Additional file 2: Table S5) for measurement of DNA methylation levels by pyrosequencing. These results confirmed those obtained using our platform and showed very high levels of methylation with high reproducibility in sperm DNA compared to the blastocyst genome (Figure 11). However, as expected there was not any linear correlation between the EDMA fold changes for the selected DMRs and their corresponding measured fold changes after the pyrosequencing confirmation (see Additional file 1: Figure S3).

## Discussion

### Development of EDMA

The study of the epigenome has become a prime focus in the effort to understand the expression of complex phenotypes such as obesity, diabetes [29, 30], mental disorders [31, 32] and cancers [33–36]. Vast epigenetic erasure and reprogramming events during early embryogenesis make this phase of development a window for perturbations, potentially having long-term impact on phenotype in adulthood. Methods for monitoring candidate loci using as little as single cell [37], one oocyte [38] or single cell blastomere [39] have been developed. The aim of the present work is to develop a robust technological platform to study genome-wide DNA methylation, suitable for the small amount of sample material obtainable from early embryos and to complement this platform with a comprehensive suite of tools for quality control and data analysis. The EmbryoGENE DNA methylation analysis (EDMA) platform was developed for compatibility with our previously published transcriptome analysis platform [1] with the aim of integrating data of both types from the same sample.

Among the three sample treatment options currently employed to study genomic DNA methylation [26], we opted for methyl-sensitive restriction enzymes (MSREs) over bisulfite conversion and affinity selection because it allowed for robust processing of DNA input of a few nanograms as well as a more straightforward data processing based on the well-established tools dedicated to microarray data analysis. An optimal procedure was sought for isolating intact genomic DNA for subsequent specific fragmentation by nucleases, which offers better repeatability than mechanical shearing (data not shown). The selection of methylated fragments thus obtained is performed by means of successive PCR reactions, while amplicon identification is achieved using a microarray, which brings the added benefit of mature data processing and analysis procedures. In addition, each microarray slide is composed of two arrays that hold two samples each (two-color arrays), allowing for the necessary inclusion of biological replicates in the experimental design. All platforms must include biological replication, since the extent of DNA methylation is naturally variable among samples of similar origin.

The procedures involved in EDMA, namely restriction endonuclease genomic fragmentation, adaptor ligation, cleavage by MSREs, selection of protected fragments by PCR and identification of fragments by microarray, are essentially those proposed for nanoHELP and for array-comparative genomic hybridization, which have been applied successfully to inputs of respectively 10 ng and a single cell [40]. Ten nanograms is the approximate quantity of DNA obtainable from 10 expanded blastocysts. Comparing 1 μg and 10 ng genomic DNA input, Oda and colleagues demonstrated that the smaller input provided higher reproducibility (R > 0.96), due probably to more complete cleavage, suggesting that low input of genomic DNA would likely be more suitable for MSRE-based platforms [41]. Sample processing used in EDMA was tested with lower gDNA inputs as low as 15 cells and genomic coverage was measured using the 50 K bovine SNP Chip which showed important loss of information due to allele drop-out when input were lower than 1 ng (data not shown). When dealing with single cell DNA methylation analysis, partial sample loss is often observed. This loss may not be problematic when aiming for targeted loci but is definitely problematic for genome-wide profiling. As such, 10 ng was set the minimal input leading to a robust DNA methylation profiling.

In order to prevent the introduction of false positives that would arise from incomplete cleavage of sample DNA [42, 43], spiked-in controls were designed to account for extent of cleavage both during the initial *MseI* fragmentation and by MSREs. Samples were tested before fragment amplification by PCR to ensure that cleavage was sufficient. When cleavage is incomplete, the MSREs treatment can be repeated. For each MSRE, a pair of synthetic DNA controls was designed, one of which was methylated *in vitro* and used as a control for fragment protection, while the unprotected counterpart acted as the cleavage target.

One of the main concerns with any genome-wide approach is the actual genomic coverage. Since the *MseI* recognition site is T/TAA, CpG dinucleotides are left intact and most CpG islands are conserved [44]. These sites are very abundant throughout the genome, resulting in small fragments well suited for LM-PCR [40, 45, 46]. By fragmenting the entire genome in this manner, methylation status outside CpG islands can be queried. This provides valuable information, since evidence is mounting for potential important roles for 5mC in non-CpG islands, which have been found more dynamic than CpG islands with respect to methylation-based regulation [47]. This is potentially even more important in early embryos where overall demethylation just occurred.

Since detection based on restriction enzymes is limited to fragments bearing the recognition sites [11, 48], adequate genomic coverage by MSRE requires a combination of nucleases [27, 44]. *In silico* analysis showed that compared to *HpaII* alone, the combination of *HpaII*, *HinP1I* and *Aci1I* increased the coverage of CpGs sites in the probes designed for EDMA from 2.3% to 8.6% and genomic coverage from 4.8% to 6.1% (see Additional file 2: Table S2). This coverage accounts for a little more than 2.3 M CpG sites, which corresponds to about 10% of all CpG sites in the bovine genome. Similar coverage is obtained using reduced-representation bisulfite sequencing (RRBS), which has been shown to be efficient with small DNA input [19, 49, 50]. Both EDMA and RRBS have several steps in common, including genomic fragmentation by restriction enzyme, adaptor ligation and PCR amplification. The main benefits of reduced-representation bisulfite sequencing are that it allows quantitative evaluation of methylation at single-base resolution and is applicable to all species since it is based on DNA sequencing, which does not require a priori knowledge of the genome [51]. However, EDMA similar to any other MSREs enrichment-based approaches generate a list of DMRs ranked in order of significance but does not provide information regarding the extent of DNA methylation which must be determined by targeted pyrosequencing. Furthermore, enrichment-based approaches using antibody or methyl-binding proteins have not been thoroughly tested with samples containing only a few ng of DNA. With larger DNA input, these methods have been shown to provide moderate resolution, since the capture depends on fragment methylation density [11, 52].

While epigenome-wide association studies and the development of technological platforms suitable for low input DNA are broadening in scope, improvement to the standardization of experimental assays across samples and to data analysis and interpretation remains slow [53]. Although amplicons obtained by LM-PCR could be processed into sequencing libraries, we opted for microarray-based identification, which is more restrictive (being limited to the probes printed on the array) but has the major benefit of compatibility with an established, robust and user-friendly data analysis pipeline. These features may limit genomic coverage but definitely increase sample turnover rate by decreasing the time required to interpret data.

In comparison to gene expression, which is most often limited to the study of protein encoded transcribed elements which account for little more than 1% of the eukaryote genome, profiling overall DNA methylation considers a vastly more complex diversity of sequences, which in turn complicate data analysis. We therefore sought to support data interpretation by binning the information according to genomic features. Such an approach increases the statistical power to identify subtle alterations in genome regions by avoiding P value dilution, through multiple-testing corrections that include the vast majority of regions considered *a priori* unlikely to be differentially methylated [54]. The data analysis pipeline built in to our platform accounts for: i) site specificity (e.g. promoter, intron, and exon), ii) region (e.g. shore, shelf, open-sea) and iii) sequence composition (e.g. CpG island density and length). This is in accordance with recent recommendations for epigenome-wide association studies [53].

The enrichment categorization implemented in the data analysis pipeline is based on previous studies [49, 55–60] and provides a full complement of graphic outputs. Even though the graphs might not all be relevant to all users, depending on the experimental design and biological hypothesis, are all produced automatically at no additional cost in time or resources on the part of the user. Overall, EDMA was developed as a cost-effective standardized platform that robustly profiles DNA methylation across the entire bovine genome. This sample processing platform is also complemented with bioinformatics supports for data analysis with the specific aim to aid data interpretation.

### The importance of genome-scale parallel analysis of the DNA methylome and transcriptome

Current technological advances and the exponential growth of epigenetics studies in the past few years, in particular genome-wide studies, are advancing our knowledge and providing more evidence for the interdependence of epigenetic and genetic variations [53]. By providing genome-wide parallel survey of the DNA methylome and the transcriptome for the same sample, EDMA offers a powerful tool for revealing highly relevant targets and potential associations between the DNA methylome and the transcriptome. In addition, overlaying methylomic and transcriptomic data has been shown to provide complementary information [60].

### Genome-wide profiles of bovine sperm and blastocyst DNA methylomes

As proof of concept, we compared the DNA methylation profiles of bovine sperm and blastocysts and performed validation by pyrosequencing on selected DMRs. All selected candidates were found to be substantially more highly methylated in sperm DNA, corroborating the results obtained with EDMA. Physiologically relevant data were also generated and compared to the current literature. The sperm genome was found hyper-methylated compared to the embryo, which is consistent with the de-methylation process known to occur after fertilization in bovine [61] and other species such as mouse [49, 50] and zebra fish [62]. The data also indicated that large numbers of loci methylated in bovine embryos are also methylated in sperm DNA, which also corroborates a previous report [62]. As previously observed in mice [50], we found that from the DMRs enrichment, that short length/low density CpG islands showed a higher tendency to changes (*i.e.* being a DMR) than other regions. The direction of change is predominantly toward hypermethylation in sperm, but not always. In addition, we observed a high level of methylation in many repetitive elements, which is also in agreement with the patterns observed in mice [49, 50].

We observed marked differences (more hypermethylated DMRs in sperm) in the extent of methylation in long-terminal-repeat (LTR) retrotransposon, similar to previous findings with mice [50]. Furthermore, it has been shown that the sperm contributed DMRs in pre-implantation embryos at LTRs were associated with reduction of DNA methylation and the most drastic methylation changes (reduction) in the sperm-to-zygote transition observed in some families of LINE and LTR retrotransposon [49]. In our previous report, we also observed a transition in DNA methylation in LTR during bovine embryo development from Day 7 to Day 12 [27].

The reason for the observed large difference in LTR methylation during early development and, as shown specifically in this study, between sperm and blastocysts is not yet clear, although these differences might reflect the importance of *de novo* establishment of genome-wide methylation in the bovine early embryo. In this regard, robust LTR retrotransposon up-regulation has been shown during activation of the bovine embryo genome [63] and found to be a general requirement for progression through to the cleavage stage in mouse embryos [64, 65]. However, the nature of its role in mammalian early development remains elusive. Furthermore, it has been suggested that LTR re-methylation occurs in the early, rather than late, pre-implantation mouse embryo [64]. Furthermore, the observation that a higher number of DMRs are present in low-complexity simple repeats in bovine blastocysts, in comparison with sperm, might represent notable dynamic changes in 5 mC in these specific class of repetitive elements during bovine early embryo development and differentiation [66].

## Conclusion

By developing EDMA, we are providing a unified and reliable approach to analyze small amounts of genomic DNA, one that offers a good balance between genomic coverage and data turnaround time. The use of a microarray for fragment identification is robust, efficiently minimizing sample-to-data time. The built-in data analysis pipeline provides efficient means for data interpretation. The integrated data analysis pipeline could be a good option for researchers with limited bioinformatics resources. The platform is at the present time specific for the bovine genome, but a similar approach could be adapted easily to any species of which the entire genome is known. Such platforms enable the study of the potential epigenetic risks associated with assisted reproductive technologies (ART) or to highlight the sequence of events occurring during the establishment of embryonic cell lines.

## Methods

### Ethics statement

Experiments took place in compliance with the guidelines of the Canadian Council on Animal Care and supervised by the Animal Protection Committee of Université Laval. These guidelines are strictly followed by the local abattoir and L’Alliance Boviteq who provided all the tissues and samples. The study did not require handling animals on university premises.

#### Microarray design

The design of the EmbryoGENE (http://embryogene.ca) DNA Methylation Array (EDMA) was based on a compilation of methylation-sensitive genetic loci found previously to be involved in early embryonic development [27]. To maximize genomic coverage, all probes on the EDMA array were designed on the assumption that genomic cleaving using the *MseI* (T/TAA) restriction endonuclease would be nearly complete. All probes therefore targeted a specific *MseI-MseI* fragment within the bovine genome. Target loci were selected on the basis of their proximity to either the putative sites identified in our previous study [27] or to known CpG islands. Tiling of fragments neighboring the selected loci was then carried out until enough loci were selected to fill one Agilent SurePrint 1×1M slide (Agilent Technologies). Test hybridizations were carried out, and the best-performing 400,000 probes were selected for placement on the final Agilent SurePrint 2×400K array. Probe quality was measured by assessing sequence specificity and signal strength variation across the set of test hybridizations. The final EDMA array (EDMA.V2) contains 414,566 probes targeting 359,738 loci, surveying 20,355 genes and 34,379 CpG islands. The microarray also contains 10,388 control probes accounting for 2.5% of all the total probes, representing 5,610 Agilent proprietary spiked-in controls, 4,634 genomic cleavage controls and 144 EDMA spiked-in methylation controls. Controls were designed with an *MseI* restriction site at their center and were tiled at every 1 M base pairs throughout the bovine genome. These were used to assess the degree of genomic digestion. The EDMA spiked-in controls are exogenous DNA fragments (*Solanum lycopersicum*) chosen for their lack of homology with the bovine genome and for the presence of specific *HpaII*, *AciI* and *HinP1I* restriction sites within their sequence. They were artificially methylated or left unmethylated to provide positive and negative controls for the methylation-sensitive cleavage. Probe design for the EDMA microarray was carried out by Genotypic Inc. (Bangalore, India). The arrays were printed using the SurePrint technology (Agilent Technologies). The details of the EDMA platform array have been deposited in NCBI’s Gene Expression Omnibus (GEO) (http://www.ncbi.nlm.nih.gov/geo/) and are accessible through GEO, Platform accession number: GPL18384.

#### Sample production and genomic DNA extraction

Bovine blastocyst production in synthetic oviduct fluid (SOF) media was performed as described previously [27]. The ovaries were collected at a local abattoir which is complying with the guidelines provided by the Canadian Council on Animal Care. Only expanded-blastocyst-stage embryos were collected, in four pools of ten embryos (*n =* 40). All embryos were washed three times with RNAse-free phosphate-buffered saline (PBS) prior to snap-freezing and storage at −80°C. Extraction of genomic DNA was carried out using an AllPrep DNA/RNA Mini Kit (QIAGEN, Mississauga, ON, Canada), and samples were eluted in 30 μL. Straws containing frozen semen from Holstein bulls were obtained from L’Alliance Boviteq Inc (Saint-Hyacinthe, QC, Canada). L’Alliance Boviteq Inc is a commercial service provider also complying with the guidelines provided by the Canadian Council on Animal Care. Sperm genomic DNA was extracted using lysis buffer, followed by ethanol precipitation. The quality and quantity of extracted DNA were analyzed by optical absorbance using a Nanodrop ND-1000 spectrophotometer (NanoDrop Technologies Inc.) and by electrophoresis on 0.5% agarose gel at 45 volts for 2 h.

#### Sample treatment

The same DNA input (10 ng) was used for both embryo and sperm samples. For quality control purposes, all samples were spiked in with a mixture of six synthetic DNA constructs harbouring *MseI* restriction sites at each end. One pair of constructs was designed for each methyl-sensitive restriction enzyme (*HpaII*, *HinP1I* and *Aci1I*) to be used in the fragment selection process. For each pair, one control DNA fragment was methylated *in vitro* using CpG methyltransferase *M.SssI* (New England Biolabs). The difference between the extents of cleavage obtained with each pair was used to calculate cleavage efficiency. Residual *MseI* sites within the fragments were measured after processing to determine the extent of genomic fragmentation.

Sample (in 30 μL of elution buffer) plus 0.5 μL of bovine serum albumin (New England Biolabs), 5 μL of 10× Buffer 4 (New England Biolabs) and 28 μL of DNAse/RNAse free water were divided into two equal fractions and fragmented using 10 U of *MseI* (New England Biolabs) as follows, 16 h at 37°C followed by 65°C for 20 min. The two fractions were combined and DNA was concentrated by ethanol precipitation following addition of 5 μg of linear acrylamide solution (Ambion) as carrier and 5 μL of sodium acetate buffer (3 M, pH 5.5, Ambion Inc.). The pellet was washed twice with 70% ethanol, air-dried and resuspended in 5 μL of DNAse/RNAse free water*.*

##### Fragmented genome DNA adapter ligation

Adaptors were ligated to the *MseI* digested genomic fragments as described previously [40]. Briefly, 5 μM of each primer (MseLig 21: 5′-AGT GGG ATT CCG CAT GCT AGT-3′, MseLig 12: 5′-TAA CTA GCA TGC-3′, IDT DNA), 0.5× One-Phor-All plus Buffer (Pharmacia Biotech) and 1.5 μL of nuclease-free water were added to the sample. Annealing was initiated at 65°C for 1 min, and the temperature was ramped at 1°C/min down to 15°C. T4 DNA ligase (5 units, Boehringer Mannheim) and 10 nmol of ATP were added and the reaction and incubated for 16 h at 15°C.

##### *HpaII*tiny fragment enrichment by ligation-mediated PCR (HELP) cocktail cleavage of ligated genomic DNA

The ligated sample was triple-cleaved with FastDigest™ methyl-sensitive restriction endonucleases (MSRE) *HpaII* (C/CGG), *HinP1I* (GC/GC), *Aci1I* (C/CGC) (Fermentas Thermo Fisher Scientific) using a sequential digestion. Samples were first digested in 50 μL reactions that included 10 μL of sample, 0.5 μL of *HpaII*, 0.5 μL of *HinP1I*, 5 μL of 10× FastDigest buffer and 34 μL of nuclease-free water for 12 h at 37°C. A second cleavage was performed by adding 0.5 μL of *Aci1I,* 5 μL of 10× FastDigest buffer and 44.5 μL of nuclease-free water and incubating at 37°C for 4 h, followed by thermal inactivation at 85°C for 10 min.

##### Verification of cleavage: qPCR of spiked-in templates

The extent of cleavage by MSREs was determined using qPCR detection of spiked-in controls. A master mixture (19 μL) containing 2 mM MgCl_2,_ 1× LightCycler FastStart DNA Master SYBR Green mix (Roche Diagnostics Canada, Laval, QC, Canada), 14.4 μL of nuclease-free water and 1 μL of cleaved sample (template) was divided into three fractions to which 0.25 μM of each forward and reverse primer designed to target the appropriate control template was added (see Additional file 2: Table S1). Each qPCR run also included a positive control (non-digested spiked-in, 1/1000 dilution) and negative control (no template). The qPCR conditions were as follows: initial denaturation was carried out at 95°C for 10 minutes, followed by 50 amplification cycles at 95°C for 5 seconds, 52°C for 5 seconds and 72°C for 20 seconds. Melting curve analysis was performed for 1 cycle with a ramp rate of 0.2°C per second, starting at 94°C for 5 seconds, 72°C for 30 seconds, and back to 94°C for 0 seconds, and cooling at 40°C.

Amplicon specificity was determined from the shape of the melting curve and the difference in cycle threshold (Δ Ct) between methylated (MSRE cleavage protected) and unmethylated (MSRE cleavage unprotected) templates was used to calculate cleavage efficiency. The DNA samples were then precipitated by ethanol, washed and dissolved in 10 μL of nuclease-free water.

##### Fragment selection by ligation-mediated PCR

Selective amplification of methylated fragments was performed using two rounds of ligation-mediated PCR (LM-PCR) as described previously [40], with some modifications. The first LM-PCR amplification was carried out in 50 μL using 10 μL of sample to which 1 μM of primers (MseLig12 and MseLig21) was added, 0.1× One-Phor-All plus Buffer (Pharmacia Biotech), 0.6× Ex Taq™ buffer without Mg^2+^ (TaKaRa), 0.1 U Ex Taq™ enzyme (TaKaRa), 1.5 mM MgCl_2_ , 0.4 mM dNTP , and 23.5 μL of nuclease-free water. The mixture was then subjected to thermal cycling as follows: 94°C (40 sec), 57°C (30 sec) and 72°C (1 min 15 sec) for 15 cycles; 94°C (40 sec), 57°C (30 sec) and 72°C (1 min, 45 sec) for 34 cycles; and 94°C (40 sec), 57°C (30 sec) and 72°C (5 min) for the final cycle. The PCR products were resolved on 1% agarose gel to assess the quality.

To obtain sufficient DNA for downstream processing, three PCR reactions were carried out using 0.75 μL aliquots from the first LM PCR, to which 1.5 μM MseLig21 primer was added, plus 1× Buffer 1 (Roche/Boehringer Mannheim, Expand Long Template), 0.2 mM dNTPs, 0.8 μL of Expand Long Template Enzyme Mix (Roche/Boehringer Mannheim) and 38.7 μL of nuclease-free water. The following four-step programs were used: 94°C (60 sec), 65°C (30 sec), and 72°C (2 min) for 1 cycle; 94°C (40 sec), 65°C (30 sec) and 72°C (90 sec) for14 cycles; 94°C (40 sec), 65°C (30 sec) and 72°C (2 min) for 9 cycles and 72°C (5 min) as the final cycle. The quality and concentration of DNA were evaluated as described above.

##### Adaptor removal

The amplified product from the second LM-PCR was purified using a QIAquick PCR Purification Kit (Qiagen) according to the manufacturer’s instructions with minor modifications. Final elution volume was 41.5 μL, of which 1.5 μL was used for quantification by absorbance measurement. The rest was divided in two separate 20 μL aliquots. Adaptors were removed by digestion for 16 h at 37°C with 1 U of *MseI* (New England Biolabs). The mixture also contained 1× bovine serum albumin (New England Biolabs, Ipswich, MA, USA), 1 × Buffer 4 (New England Biolabs), and 24 μL of nuclease-free water. The reaction was terminating by heating for 20 min at 65°C. The sample was then purified using a QIAquick PCR Purification Kit (Qiagen), eluted in 23.5 μL of nuclease-free water and quantified by absorbance.

##### Sample labeling and hybridization

For each sample, 2.5 μg of DNA was labeled using the Universal Linkage System (ULS) labeling kit (Kreatech Biotechnology) according to the manufacturer’s instructions with minor modifications: 1 μL of Cy-ULS dye was added per 1 μg of genomic DNA adjusted with 10× labeling buffer. The labelling mixture was then held for 30 min at 85°C in a thermocycler, followed by 3 min on ice. Non-reacted ULS-Cy3/5 was removed by purification using a QIAquick PCR Purification Kit and samples were eluted in 23.5 μL nuclease-free water. A 1.5 μL aliquot was used to determine DNA concentration and dye incorporation using the ND-1000 NanoDrop. Hybridizations were performed according to the microarray manufacturer’s instructions (Agilent Technologies). Briefly, 1 μg of labeled sample (in 40 μL) was mixed with 158 μL of hybridization master mix containing 25 μL of bovine Cot-1 DNA (1.0 mg/mL, Bovine Hybloc competitor DNA, Applied Genetics Laboratories), 2.6 μL of Agilent 100x Blocking Agent and 130 μL of Agilent 2× HI-RPM Hybridization Buffer (Agilent Technologies). Samples were held at 95°C for 3 min and at 37°C for 30 min followed by addition of 65 μL of Agilent-CGHBlock (final volume 260 μL). The samples were loaded onto the microarray and hybridization was carried out in a hybridization oven (Shel Lab) for 40 h at 65°C and 20 rpm. Washing was carried out according to the microarray manufacturer’s instructions and slides were scanned with the PowerScanner (Tecan) and analyzed with Array-Pro Analyzer 6.3 software (MediaCybernetics).

#### Bioinformatics

##### Data analysis pipeline

To complement our transcriptomic platform [1], a complete suite of data analysis tools was added and integrated into the EmbryoGENE LIMS and Microarray Analysis (ELMA) gateway (http://elma.embryogene.ca/). This pipeline processes sample-associated information, experimental design and protocols, as well as microarray data analysis for identification of differential gene/loci lists and further data mining for enrichment analyses. EDMA data was analyzed using the Limma package from Bioconductor [67, 68]. First, Loess intra-array normalization followed by quantile inter-array scale normalization were applied. Normalized data was then fitted to a linear model and Bayesian statistics of differential expression were obtained. Differences in DNA methylation were considered significant when the P value was < 0.05 and the absolute log2 fold-change was at least 1.5. The inter-treatment comparisons were conducted using between-group analysis based on Eigen values using the Bioconductor package MADE4 [69]. Visualization tools were developed to generate plots of the extent of cleavage of spiked-in controls and overall genomic cleavage for quality control purposes. Different plots including Volcano plots were generated to visualize the amount of differentially methylated regions among treatments. Normalized data and a list of differentially methylated loci can be exported as text files for downstream data mining. Different enrichment analyses for genome-scale DNA methylation data are performed through a string of integrated scripts that categorize the information based on CpG island density [49], CpG island length [57], CpG island distance [58], genomic location and types of repetitive elements [70]. The pipeline also pinpoints methylation hot spots, generates bedgraph files appropriate for visualization within a genome browser, and uses Circos plot [71] to generate a circular graph representing overall methylation levels and correlating these to transcription levels, if the latter data are available.

##### Parameters definitions

The CpG island lengths, density and positions for the UMD3.1 (ftp://ftp.cbcb.umd.edu/pub/data/assembly/Bos_taurus/) build of the bovine genome was obtained from the UCSC genome browser [72]. For any given gene in EDMA, there are five types of annotation “windows” including; 1) Distal promoter, 2) Promoter, 3) Proximal Promoter, 4) Exons and 5) Introns. The “Proximal Promoter”, “Promoter” and “Distal Promoter” regions are defined as the first 1 kbp, 5 kbp and 50 kbp 5′ of the transcription start site (TSS). For each probes, genes are added to the appropriate columns if their *MseI*-*MseI* fragment overlap those windows. Genomic locations which were not part of a CpG island were further split into three types based on their distance from the nearest CpG island: “CpG shores” for regions within 2,000 nucleotides of an island, “CpG shelves” for regions between 2,000 and 4,000 nucleotides of an island and “Open Sea” for regions further away [58]. Probes whose fragment overlaps a CpG island were annotated using the descriptive characteristics of that island. Of all CpG island surveyed by the array, those in the bottom 20 percentile of length were classified as “short”, those at the top 80 percentile were classified as “long” and all others were classified as “intermediate” [57]. The same scheme was applied to CpG island density, with the bottom 20 percentile being labelled “low density”, the top 80 percentile being labelled “high density”, and all others being labelled “Intermediate density” [49]. Bovine repetitive elements content were identified by RepeatMasker (http://www.repeatmasker.org/) with build 20120418 of the RepBase database [70] and then used the repeat classes attribute (LINE, SINE, etc.) as a basis for category enrichment. The “methylation hot spots” is calculated as the averages p-values of differential methylation over windows of 100 K nucleotides. More specifically, for all probes on the array, we look up all other probes within 100 K nucleotides upstream and downstream, and average the p-values thus obtained. The averaged p-values have no statistical meaning, but can be used as an indicator for regions of interest, which we called a “methylation hot spot”.

### Data validation

Pyrosequencing was performed as described previously [73]. Briefly, blastocyst and sperm DNA samples were bisulfite-converted using an EZ DNA methylation direct KIT, (ZYMO Research) followed by amplification of target loci by PCR prior to sequencing. For all pyrosequencing assays, PyroMark Assay Design software (Qiagen) was used to design three oligonucleotide primers (forward, reverse and sequencing), synthesized by Integrated DNA technologies (Coralville). All reverse primers were biotinylated at the 5′ terminus and purified by HPLC. PCR amplification was carried out in 25 μL containing 0.2 μM of each forward and reverse 5′-biotinylated primer, 1.25 U of Platinum Taq DNA polymerase (Invitrogen), 1× Taq DNA polymerase buffer, 0.2 mM dNTPs, 3–4 mM MgCl_2_, 2 μL of bisulfite-treated DNA and 17.5 μL of nuclease-free water. The following three-step program was used: 95°C (5 min) for 1 cycle, 95°C (30 sec), 48°C for DMR1b, 49°C for DMRs 2 and 3a, 50°C for DMRs 1a, 3b, 5–8 and 54°C for DMR 4 (30 sec) and 72°C (30 sec) for 35 cycles, and finally 72°C (5 min). The specificity of the amplification was verified by electrophoresis on 2% w/v agarose gel at 90 V for 45 min. Templates were purified by adding 2 μL of streptavidin-coated Sepharose beads (GE Healthcare) in the presence of 40 μL of binding buffer (Qiagen) and then pyrosequenced [74]. Pyrosequencing reactions were conducted using a PyroMark Q24 apparatus (Qiagen).

### Availability of supporting data

The data analysis pipeline user manual is available at; [EDMA user manual]: http://emb-bioinfo.fsaa.ulaval.ca/bioinfo/html/epigenetics/Epigenetics%20Analysis%20Pipeline.pdf.

The dataset of microarray results have been deposited in NCBI’s Gene Expression Omnibus (GEO) and are accessible through GEO series accession number: GSE57709 (http://www.ncbi.nlm.nih.gov/geo/query/acc.cgi?acc=GSE57709).

## Electronic supplementary material

Additional file 1: Figure S1: (A) This histogram shows the *in silico* analysis of the lengths of *MseI/MseI* fragments across the bovine genome. Higher frequencies of shorter fragments with an average size <160 bp are observed. (B) The Venn diagram shows the overlaps between restriction sites of the HELP cocktail MSREs within the genomic *MseI* fragments targeted by EDMA probes. **Figure S2.** This figure shows the histogram number of (A) CpG dinucleotide per *MseI* restriction fragments and histograms number of MSREs ((B) *HpaII;* (C) *HinP1I;* (D) *AciI)* restriction sites per restriction fragments in EDMA. **Figure S3.** This figure clearly shows that there is not any correlation between the determined EDMA fold change and Pyrosequening results due to the enrichment-based nature of the applied protocol. (PDF 478 KB)

Additional file 2: Table S1: The designed Primers for the MSRE digestion quality control step. **Table S2.** Genomic and CpG coverage by *MseI* fragments targeted by EDMA probes as a function of the MSRE sites present within those fragments. **Table S3.** Gene and CpG Island coverage by EDMA probes. **Table S4.** Breakdown of the location of EDMA probes in relation to annotated features of the bovine genome. **Table S5.** The properties of the selected hypermethylated DMRs and their primers designed used for pyrosequencying. (PDF 497 KB)

## Abbreviations

ART: Assisted reproductive technologies
DMR: Differentially methylated region
EDMA: EmbryoGENE DNA methylation analysis
ELMA: EmbryoGENE LIMS and microarray analysis
HELP: *HpaII* tiny fragment enrichment by ligation-mediated PCR
LINE: Long interspersed element
LM-PCR: Ligation-mediated PCR
LTR: Long terminal repeat retrotransposons
MSRE: Methyl-sensitive restriction endonuclease
ncRNA: Non-coding RNA
NNAT: Neuronatin gene
PEG10: Paternally expressed 10 gene
QC: Quality control
RRBS: Reduced-representation bisulfite sequencing
SINE: Short interspersed element.
