# Supplementary material for: An integrated platform for bovine DNA methylome analysis suitable for small samples
Source: BMC Genomics. 2014 Jun 9;15(1):451. doi: 10.1186/1471-2164-15-451 (PMC4092217; doi:10.1186/1471-2164-15-451)
Supplement: Supplementary file 1 — Additional file 1: Figure S1: (A) This histogram shows the in silico analysis of the lengths of MseI/MseI fragments across the bovine genome. Higher frequencies of shorter fragments with an average size <160 bp are observed. (B) The Venn diagram shows the overlaps between restriction sites of the HELP cocktail MSREs within the genomic MseI fragments targeted by EDMA probes. Figure S2. This figure shows the histogram number of (A) CpG dinucleotide per MseI restriction fragments and histograms number of MSREs ((B) HpaII; (C) HinP1I; (D) AciI) restriction sites per restriction fragments in EDMA. Figure S3. This figure clearly shows that there is not any correlation between the determined EDMA fold change and Pyrosequening results due to the enrichment-based nature of the applied protocol. (PDF 478 KB) [file 12864_2014_6213_MOESM1_ESM.pdf]

Supplementary Figures

A

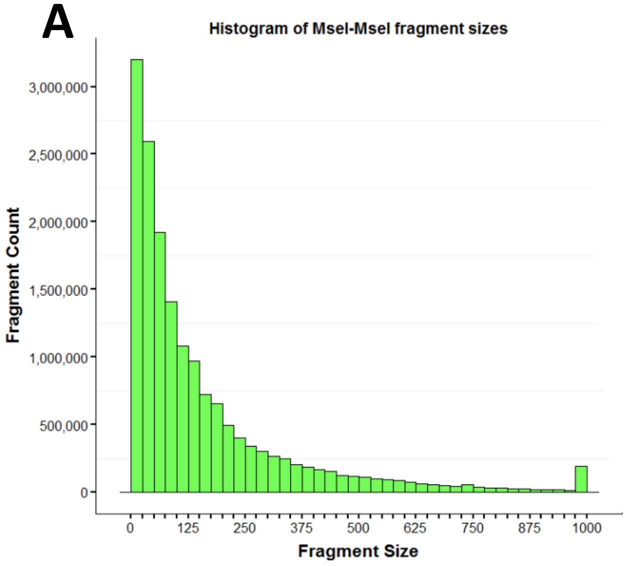

B

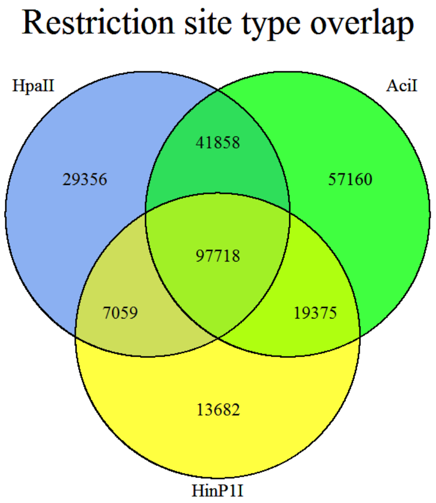

Figure S1. (A) This histogram shows the *in silico* analysis of the lengths of *MseI/MseI* fragments across the bovine genome. Higher frequencies of shorter fragments with an average size <160 bp are observed. **(B)** The Venn diagram shows the overlaps between restriction sites of the HELP cocktail MSREs within the genomic *MseI* fragments targeted by EDMA probes.

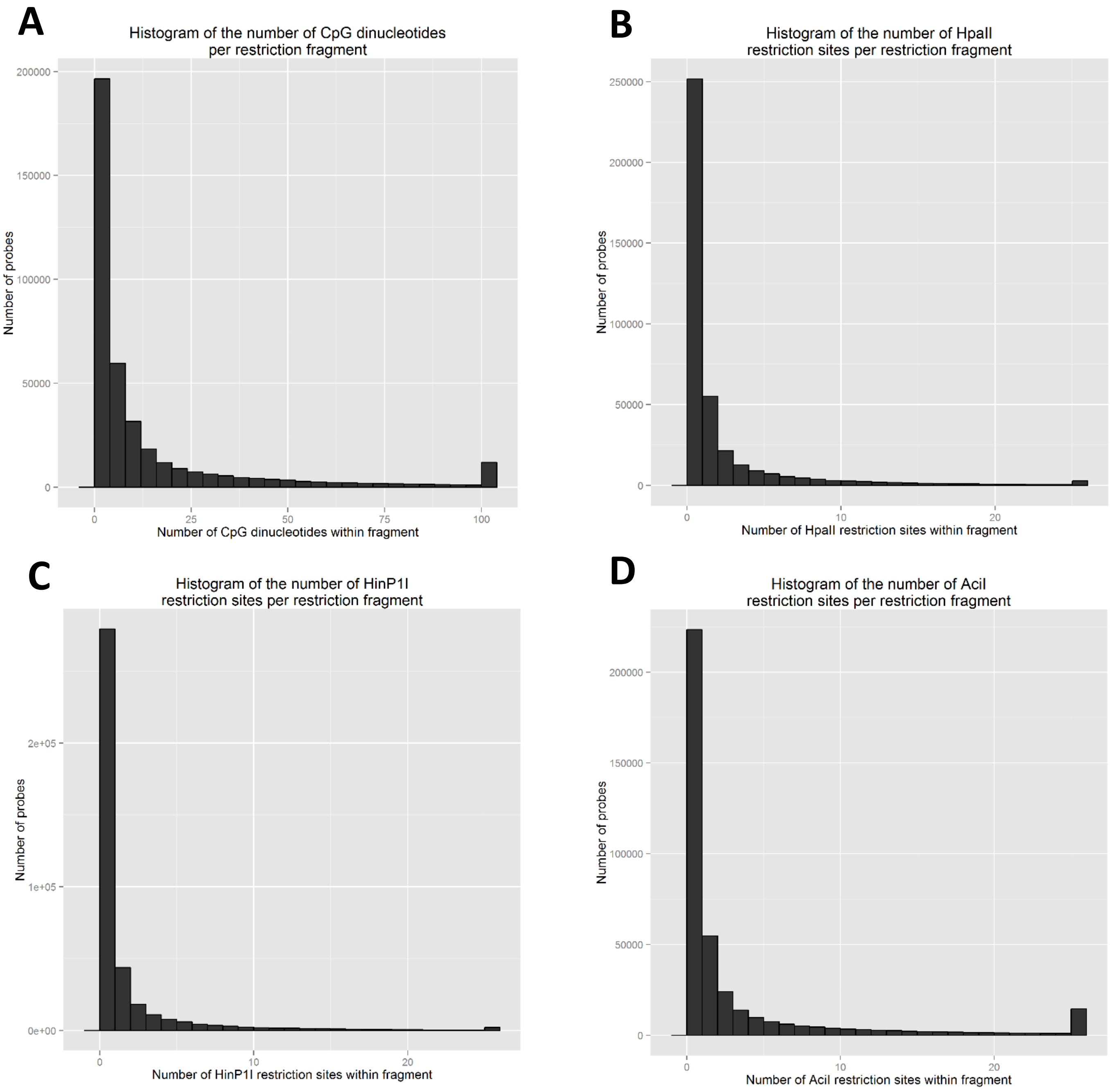

Figure S2. This figure shows the histogram number of **(A)** CpG dinucleotide per *MseI* restriction fragments and histograms number of MSREs (**(B)** *HpaII;* **(C)** *HinP1I;* **(D)** *AciI)* restriction sites per restriction fragments in EDMA.

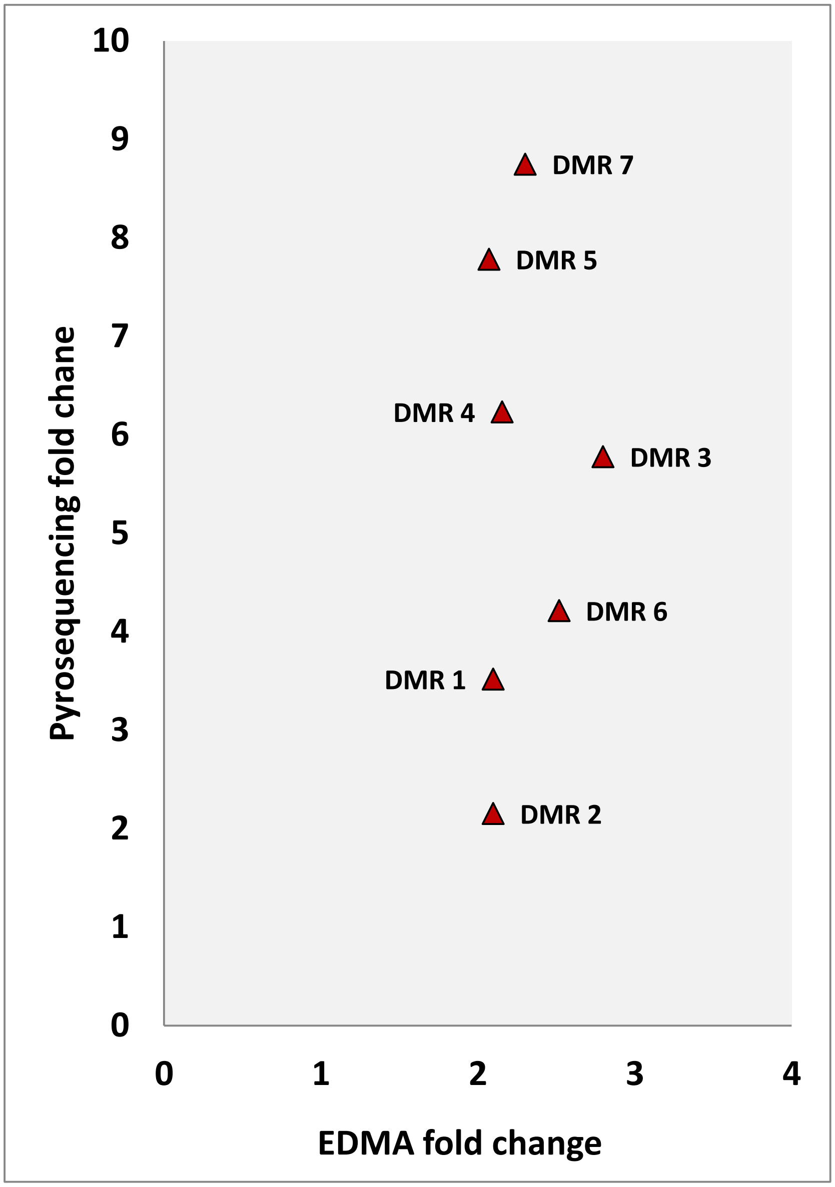

Figure S3. This figure clearly shows that there is not any correlation between the determined EDMA fold change and Pyrosequening results due to the enrichment-based nature of the applied protocol.
