# Supplementary material for: An integrated platform for bovine DNA methylome analysis suitable for small samples
Source: BMC Genomics. 2014 Jun 9;15(1):451. doi: 10.1186/1471-2164-15-451 (PMC4092217; doi:10.1186/1471-2164-15-451)
Supplement: Supplementary file 2 — Additional file 2: Table S1: The designed Primers for the MSRE digestion quality control step. Table S2. Genomic and CpG coverage by MseI fragments targeted by EDMA probes as a function of the MSRE sites present within those fragments. Table S3. Gene and CpG Island coverage by EDMA probes. Table S4. Breakdown of the location of EDMA probes in relation to annotated features of the bovine genome. Table S5. The properties of the selected hypermethylated DMRs and their primers designed used for pyrosequencying. (PDF 497 KB) [file 12864_2014_6213_MOESM2_ESM.pdf]

Supplementary Tables

**Table S1. The designed Primers for the MSRE digestion quality control step.**							
Solanum lycopersicum selected genes	**MSRE[a]**		**Spike-in (QC)[b] primers**	**Sequence**	***GC%***	***Length***	***Temp. (^{o}C)[c]***
Rubisco Large subunit	***Aci1***	***Methylated***	*1-1*	5’- CATTCCGAGTAACTCCTCAACC-3’	50	22	54
			Forward/reverse	5’- CTTTTGCTAATACCCGGAAGTG-3’	45	22	
Phytochrome B1		***Non-methylated***	*9-2*	5-‘ CGGGTATGACAGGGTTATGG -3’	55	20	54
			Forward/reverse	5’- GGACCGAACAGAAGTGTGGT-3’	55	20	
			9-2 (QC primers)	5’- AGCAGAACAGGGTGAGAATGAT-3’	45	22	52
			Forward/reverse	5’- AGACCCCATATTTGCCATGT-3’	45	20	
Glutamine synthetase	***HpaII***	***Methylated***	*7-1*	5’- CAGCTGGTGATTAAGTGTGG -3’	50	20	54
			Forward/reverse	5’- TGCAATCATGGAGGTAACGA-3’	45	20	
Nitrite reductase		***Non-methylated***	*14-10*	5’- CGAGTTAATCAGGAATCCAGTTGGAAATC-3’	41	29	54
			Forward/reverse	5’- CGAGTTAATGCAAGATCATTGATATGAGG-3’	37	29	
			14-10 (QC primers)	5’- TCTGGCTGGAATTGATCCTG-3’	50	20	52
			Forward/reverse	5’- TCATAAAGATCATGAGACCCT-3’	38	21	
TOMWIPIG	***HinP1I***	***Methylated***	*18-4*	5’ - AAATCTCCCGCTTCGCCCTTAT-3’	50	22	54
			Forward/reverse	5’- CAGTTAAGCCATGAGAGTTTCAAAGGCTGTCGAT-3’	44	34	
Nitrate reductase		***Non-methylated***	*10-11*	5’- CGAGTTAATGGAGGAGAGACATGG-3’	50	24	54
			Forward/reverse	5’- CGACTTAACAACCAACTCGAAGAACCCTAC-3’	46	30	
			10-11 (QC primers)	5’- ACATGGAGTGTTCAGTTGTTG-3’	43	21	52
			Forward/reverse	5’- CGACTTACCACCCAACTCGAAGAACCCTAC-3’	53	30	

a) MSRE: Methyl-sensitive restriction endonuclease; **b)** ***QC primers:*** *Quality control primers;* ***c) Temp. (^{o}C):*** *Annealing temp.*

**Table S2. Genomic and CpG coverage by *MseI* fragments targeted by EDMA probes as a function of the MSRE sites present within those fragments**						
	**Bovine genome* (No.)**	**All probes (No.)**	**All enzymes (No.)**	***HpaII* (No.)**	***AciI* (No.)**	***HinP1I* (No.)**
**Genomic coverage of fragments**	**-**	7.5 % (200,653,194)	6.1 % (163,284,790)	4.8 % (129,228,720)	5.4 % (1,449,615,52)	4.1 % (109,590,310)
**Coverage CpG per fragments**	**-**	20.7% (5,640,598)	19.7 % (5,368,673)	17.7 % (4,808,063)	18.8 % (5,110,353)	16.4 % (4,448,586)
**Coverage CpG per enzymes**	**-**	8.6 % (2,343,216)	8.6% (2,343,216)	2.3 % (630,128)	4.6 % (1,246,095)	1.7 % (466,993)

MSRE: Methyl-sensitive restriction endonuclease.

*****The bovine genome (*Bos Taurus*) has an average genome size of 2,670,422,299 bp and 27,203,575 CpG sites in entire genome.

**Table S3. Gene and CpG Island coverage by EDMA probes.**					
	**Genes**			**CpG Islands**	
**Coverage**	Covered features (#)	Covered features (%)	**Coverage**	Covered features (#)	Covered features (%)
Genes covered (Body or promoter)	16,912	77.8%	CGIs covered	44,182	87.3%
Genes covered (Distal promoter)	20,361	93.6%	Total CGIs	50,633	**-**
Total genes	21,747	**-**			

Table S4. Breakdown of the location of EDMA probes in relation to annotated features of the bovine genome

Probe. Category	Probe. No.	Probe %
CpG Island	52,228	12.6%
4k of a CpG island	186,255	44.9%
Gene	195,580	47.2%
Gene or Its Promoter	207,527	50.1%
50k of a Gene	295,096	71.2%
Repeated Elements	217,362	52.4%

Probe Category: The probes within the specified category
Probe No: Number of Probes
Probe %: Percentage of all probes in the microarray

Table S5. The properties of the selected hypermethylated DMRs and their primers designed used for pyrosequencying.							
**DMR**[a]	**Chr.** [b]	**Bovine genomic sequence number**	**Pyrosequencing primers**	**Sequence**	***Temp.***[c]	***CpG***[d]	***Length***[e]
**1**	1	155133370-155133821	Forward<br>Reverse (5'- Biotinated)<br>Sequencing	5'- TTAGTAATTTAGATGGGGAAGTTTAAT-3'<br>5'- TCTTACCAAAAAAATTTCCAAAATAACAC-3'<br>5'- AGATGGGGAAGTTTAATT-3'	50°C	5	56
**2**	1	155133370-155133821	Forward<br>Reverse (5'- Biotinated)<br>Sequencing	5'- TGGAAATTTTTTTGGTAAGAATAGTTAAT-3'<br>5'- CTCCCCTTTAAAATAATTAAAACTTAAATC-3'<br>5'- AAGAATAGTTAATTTTATGTTTGT-3'	48°C	4	53
**3**	3	119346133-119346235	Forward<br>Reverse (5'- Biotinated)<br>Sequencing	5'-TTTTTTGTAGGAAATGGGTTTGTT-3'<br>5'- TTCCCCCTATTCTATTTCTCCTAATAA-3'<br>5'- AGGAAATGGGTTTGTTT-3'	49°C	5	61
**4**	4	118285867-118285998	Forward<br>Reverse (5'- Biotinated)<br>Sequencing	5'- TGGAGAGTTTATAAAGTTAGGGTTAGA-3'<br>5'- ATAATTCTTAAACTCCAAAAACTAATTCAC-3'<br>5'- GAGTAGGTTTTTTTTATTTTTAGAT-3'	49°C	2	22
**5**	14	27808884-27809309	Forward<br>Reverse (5'- Biotinated)<br>Sequencing	5'- GTAAGGTAGTAGGGGTTTTAGTTGAT-3'<br>5'- CTCTACTCCCTCCATCTACAAACCAAATAC-3'<br>5'- GTTTTAGTTGATGTTAAAAGTT-3'	54°C	7	57
**6**	19	32804128-32804528	Forward<br>Reverse (5'- Biotinated)<br>Sequencing	5'- GTTTGGAGAGTTAGAAGTAAAAGTTAGAT-3'<br>5'- ACCTCAAAACCTTCAAACAAATAAA-3'<br>5'- TAGGTAGGGGTGGTT-3'	50°C	4	56
**7**	21	62757397-62757852	Forward<br>Reverse (5'- Biotinated)<br>Sequencing	5'- AAAATTTAGATGGGAAGGAATG-3'<br>5'- TACAACATCCAACATCTCTCTAACT-3'<br>5'- GATGGGAAGGAATGT-3'	50°C	7	62

a) DMR: Differentially methylated region; **b):Chr.:** Chromosome number**; c):** ***Temp. (^{o}C):*** *Annealing temperature****; d) CpG:*** *The number of CpG in the pyrosequenced fragment;* ***e) Length:*** *The length of pyrosequenced fragment.*
